# REFINE (Rapid Feedback for quality Improvement in Neonatal rEsuscitation): an observational study of neonatal resuscitation training and practice in a tertiary hospital in Nepal

**DOI:** 10.1186/s12884-020-03456-z

**Published:** 2020-12-03

**Authors:** Rejina Gurung, Abhishek Gurung, Omkar Basnet, Joar Eilevstjønn, Helge Myklebust, Sakina Girnary, Shree Krishna Shrestha, Dela Singh, Laxman Bastola, Prajwal Paudel, Sandhya Baral, Ashish KC

**Affiliations:** 1 Golden Community, Jawagal, Lalitpur, Nepal; 2 Laerdal Medicine/Laerdal Global Health, Stavanger, Norway; 3 Pokhara Academy of Health Sciences, Pokhara, Nepal; 4 Paropakar Maternity and Women’s Hospital, Kathmandu, Nepal; 5 Society of Public Health Physician Nepal, Kathmandu, Nepal; 6 Department of Women’s and Children’s Health, Uppsala University, Dag Hammarskjölds väg 14B, 1 tr, 752 37 Uppsala, Sweden

**Keywords:** Newborn, Newborn resuscitation, REFINE, Helping babies breathe

## Abstract

**Background:**

Simulation-based training in neonatal resuscitation is more effective when reinforced by both practice and continuous improvement processes. We aim to evaluate the effectiveness of a quality improvement program combined with an innovative provider feedback device on neonatal resuscitation practice and outcomes in a public referral hospital of Nepal.

**Methods:**

A pre- and post-intervention study will be implemented in Pokhara Academy of Health Sciences, a hospital with 8610 deliveries per year. The intervention package will include simulation-based training (Helping Babies Breathe) enhanced with a real-time feedback system (the NeoBeat newborn heart rate meter with the NeoNatalie Live manikin and upright newborn bag-mask with PEEP) accompanied by a quality improvement process. An independent research team will collect perinatal data and conduct stakeholder interviews.

**Discussion:**

This study will provide further information on the efficiency of neonatal resuscitation training and implementation in the context of new technologies and quality improvement processes.

**Trial registration:**

10.1186/ISRCTN18148368, date of registration-31 July 2018

## Background

Worldwide, approximately 10 million newborns every year require assistance to breathe after birth. Studies show that timely and effective ventilation of these babies can significantly improve newborn survival. However, insufficient number of providers trained in newborn resuscitation and a lack of clinical tools remain significant barriers [1–3]. The American Academy of Pediatrics (AAP) and their partners developed Helping Babies Breathe (HBB), a simulation-based curriculum, to train and equip healthcare providers to evaluate and support breathing at birth [4].

However, in resource-constrained environments like Nepal, there are several challenges to neonatal resuscitation training and implementation, including lack of time, equipment and resources [5]. Singhal et al., while developing an education program for newborn resuscitation in resource-constrained settings identified that additional training, education and guidance are necessary for improve retention and assimilation of resuscitation skills in clinical practice [6].

Simulation-based training has been used increasingly to help health workers convert knowledge and skills into practice [7, 8]. Simulations help workers combine and reinforce their academic, intellectual, practical and interactive skills [9]. However, not much is known about the translation of training into clinical practice [10]. Also, health workers in poorly resourced settings may be unfamiliar with simulation-based training or even how to meet their training needs [11]. We believe that regular simulation-based practice in the form of drills will help them to translate their knowledge and skills into practice.

The Rapid Feedback for quality Improvement in Neonatal rEsuscitation (REFINE) study aims to improve the resuscitation skills of health workers using HBB training enhanced with a real-time feedback system (the NeoBeat newborn heart rate meter with the NeoNatalie Live manikin and upright newborn bag-mask with PEEP). The goal is to improve clinical performance and therefore newborn outcomes, monitored and reinforced using a quality improvement process.

## Methods/design

### Aim

The aim of this study is to evaluate the effect of an educational and quality improvement package on intrapartum-related mortality, health workers’ performance in newborn resuscitation, and stakeholder perceptions.

### Study design

This is a pre- and post-intervention observational study. The pre-intervention period July 2017 – September 2018 will be taken as baseline period. The intervention period will be October 2018 – December 2020.

### Study setting

The study will be conducted in the delivery unit at Pokhara Academy of Health Sciences (PoAHS), Pokhara. The hospital has 400 beds and 240 health workers. There is a delivery area for normal deliveries and an operation theatre for Caesarean-sections. The total annual delivery rate was 8610 in 2018, the stillbirth rate is 14 per 1000 total births with a neonatal mortality rate of 19 per 1000 live births.

### Study participants

Study participants include health care providers and babies born in the hospitals. All the health care providers working in the labour room are eligible to the study. During the orientation to the health care providers on the quality improvement project, a group consent will be taken on the observation of health care provider’s simulated practice in neonatalie live and clinical practice. Women with gestational age equal to or more than 22 weeks with a fetal heart sound admitted in the labour room for delivery will be approached for enrollment in the study. Women who consent to get enrolled will be included in the study.

### Data collection

A team of data collectors, supervised by a research manager, will collect information on mortality outcomes and health worker’s performance. The data collectors will extract information from the hospital records on socio-demographic characteristics of women enrolled and observe clinical resuscitation and immediate newborn practices. A standardized data collection protocol will be used to ensure consistency. Training will be provided to the data collectors on selection criteria, obtaining consent, clinical observation and data retrieval.

### Data retrieval

Data will be extracted from the patient records and ward registers for the full study period.

### Clinical observations

Clinical observations will be conducted to capture the clinical variables related to newborn resuscitation. A tablet-based observation tool has been developed and validated by Laerdal Global Health together with researchers from University of North Carolina and Kampala University. This novel application records newborn heart rate together with clinical variables.

### Process evaluation

A qualitative data collection process will be implemented where an independent team will conduct in-depth interviews and focus groups discussions with the health workers and relevant stakeholders (supplementary file 1). Furthermore, video filming will also be done in the end to document the implementation process and outputs.

### Outcome measure

#### Primary outcome measure

Intrapartum stillbirth or death of newborn within 24 h.

#### Secondary outcome measures

Health workers’ performance (based on clinical observations):

- ◦ Time to apply NeoBeat heart rate meter
- ◦ Time to first spontaneous breath
- ◦ Time of first inflation with bag-mask
- ◦ Ventilation with bag-mask in non-breathing babies at the rate of 40–60 breaths per minute
- ◦ Heart rate from the time of NeoBeat application until 10 min after birth
- ◦ Apgar score at 1, 5 and 10 min after birth
- ◦ Time of first spontaneous breath for non-breathing babies

### Interventions

#### The REFINE intervention package will be implemented using four different approaches

**Fig. 1:**
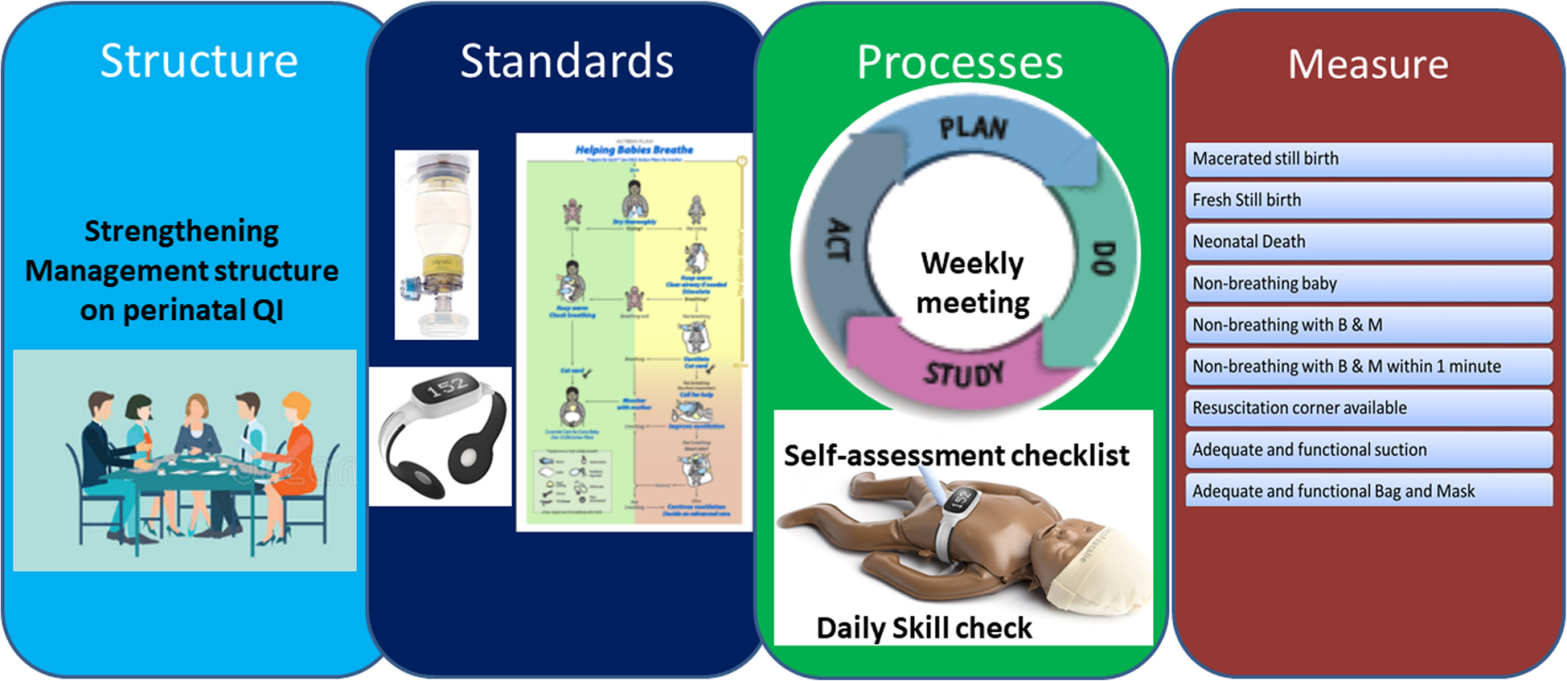
Intervention model

1. A half a day workshop will be done to review the health worker’s performance on neonatal resuscitation with hospital managers and head of department. The workshop participants will be oriented on the new technology implementation through quality approach;
2. One-day training on Helping Babies Breathe 2.0 version will be provided to health workers working in labour and delivery room;
3. A half of day orientation on the new technologies will be provided to the health workers. Health workers will be oriented on user guidance on neonatalie live, upright bag-mask with PEEP and Newborn Heart Rate Meter. Neonatalie live is a high fidelity simulator which provides real time feedback and debrief on the adequacy and ventilation practice in the simulator. Facilitation will be done to ensure that health workers practice neonatalie live before they resume their work in the labour room on a daily basis. Newborn heart rate meter measures neonatal heart rate and is placed in all babies who require resuscitation at birth. These technologies is co-developed by Laerdal Global Health.
4. A periodic meeting will be facilitated by the research team to discuss on the progress on implementation of the new technologies for improving neonatal resuscitation care using a Plan-Do-Study-Act (PDSA) process. A performance dashboard on neonatal resuscitation will be used to conduct the PDSA meetings (Fig. 1).

### Sample size and power calculation

Power calculation for the primary outcome is based on preliminary estimates of an intrapartum mortality of 20/1000 births and 8610 deliveries in the study hospitals per year. We estimate 20% reduction in the intrapartum mortality following implementation of the quality improvement package. With level of significance at 5% and statistical power at 80% to detect 20% reduction in intrapartum mortality we will require 10,023 birth. Intrapartum mortality is calculated as intrapartum stillbirth and first day mortality. Based on the current annual delivery rate, the duration of the intervention period will be 15 months.

### Data management

For the purpose of ensuring high quality data collection, management and data security an independent data monitoring committee (IDMC) will be formed. All data will be saved on a server and backed up on a weekly basis. The project manager and the data manager will make spot checks monthly to verify records with the primary source of data. A quality control team from Laerdal Global Health will provide oversight on a regular basis to ensure quality of data collection and to avoid data loss. Procedures for data storage and handling will follow strict ethical review board and confidentiality rules.

### Data analysis

**Fig. 2:**
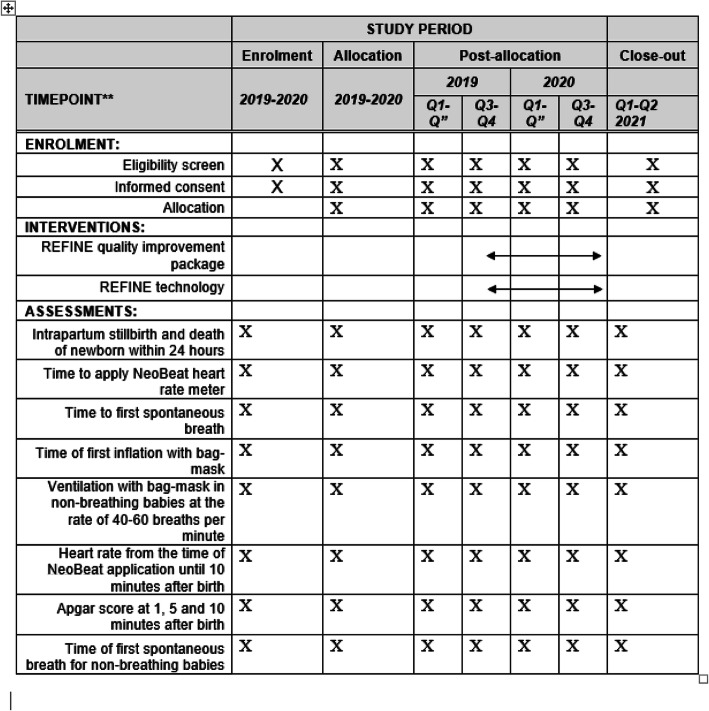
SPIRIT timeline

Analysis of the intervention package effectiveness will be based on comparison between the baseline and end line intrapartum mortality and health worker performance (Fig. 2). Cross-sectional data gathered through the established surveillance system and clinical observations will produce a large dataset allowing for epidemiological analyses. The final report of this protocol will follow the general STrengthening the Reporting of OBservational studies in Epidemiology (STROBE) Statement and figure (supplementary file 2) [12]. Process evaluation will follow Medical Research Council (MRC) framework [13] for implementation and process level data. We have filled Standard Protocol Items: Recommendations for Interventional Trials (SPIRIT) checklist required for study protocol (supplementary file 3).

### Ethical consideration

For this study, ethical approval (no. 87/2018) has been received from the national ethical review board, Nepal Health Research Council (NHRC). Ethical clearance will also be received from the Institutional Review Board (IRB) in the academy where the study will be conducted. Group consent will be taken from health workers to collect information on health worker’s performance in simulated and clinical setting. Written informed consent will be taken from the pregnant women before clinical observations and confidentiality will be maintained. All ethical principles outlined in the World Medical Association (WMA) Declaration of Helsinki will be observed [14]. Authorship will follow Vancouver principles and all funding sources will be declared in publications.

## Discussion

Helping Babies Breathe training has been successful in improving newborn outcomes [15, 16]. Previous study led by KC and colleagues has also shown that a quality improvement approach leads to improved adherence of the health workers in practicing neonatal resuscitation protocol. It also led to decreased intrapartum stillbirth and newborn mortality [17, 18]. Evidence has shown that improved newborn resuscitation knowledge and skills are necessary to decrease birth asphyxia related newborn mortalities [19]. Furthermore, it is important to understand that not all non-breathing babies require bag and mask ventilation and may breathe through simple stimulation or airway clearance [20]. These observations are vital to understanding the process and providing care.

This research team has been involved in a large-scale QI intervention in 12 public hospitals of Nepal, which aimed to improve adherence of health workers for newborn interventions aimed at improving newborn survival [21]. The current study will build upon our experience and aims to improve health workers’ capacity through further enhancements in the both training and daily practice by introducing novel resuscitation technologies. A QI approach should identify bottlenecks and help overcome barriers. The study will also evaluate the use of new technologies. The clinical, QI, and qualitative results will guide future newborn policies and protocols and related resource investments.

One of the limitations of the study can be unforseen short-term impact of the training and drills on clinical practice. This study is built upon previous and ongoing QI interventions, there is a likelihood of contamination resulting in improved outcomes. Further, the technology such as neonatalie live, neobeat and upright bag-and-mask are provided by Laerdal Global Health, we will manage the data and results anonymously maintain research integrity. Finally, if there is evidence of improved outcomes, we plan to scale-up the interventions with the goal of sustainability in the medium and long term.

## Supplementary Information

**Additional file 1.** SPIRINT checklist filled.

**Additional file 2.** STROBE flow figure.

**Additional file 3.** Indepth interview questionnaires.

## Data Availability

Not applicable.

## Abbreviations

AAP: American Academy of Pediatrics
ENAP: Every Newborn Action Plan
HBB: Helping Babies Breathe
IDMC: Independent Data Monitoring Committee
ILCOR: International Liaison on Resuscitation
IRB: Institutional Review Board
NePeriQIP: Nepal Perinatal Quality Improvement Project
NHRC: Nepal Health Research Council
PoAHS: Pokhara Academy of Health Sciences
REFINE: Rapid Feedback for quality Improvement Neonatal rEsuscitation
SDG: Sustainable Development Goal
STROBE: STrengthening the Reporting of OBservational studies in Epidemiology
QI: Quality improvement
WMA: World Medical Association
