# Supplementary material for: REFINE (Rapid Feedback for quality Improvement in Neonatal rEsuscitation): an observational study of neonatal resuscitation training and practice in a tertiary hospital in Nepal
Source: BMC Pregnancy Childbirth. 2020 Dec 3;20:756. doi: 10.1186/s12884-020-03456-z (PMC7712979; doi:10.1186/s12884-020-03456-z)
Supplement: Supplementary file 2 — Additional file 2. STROBE flow figure. [file 12884_2020_3456_MOESM2_ESM.pptx]

## Slide 1
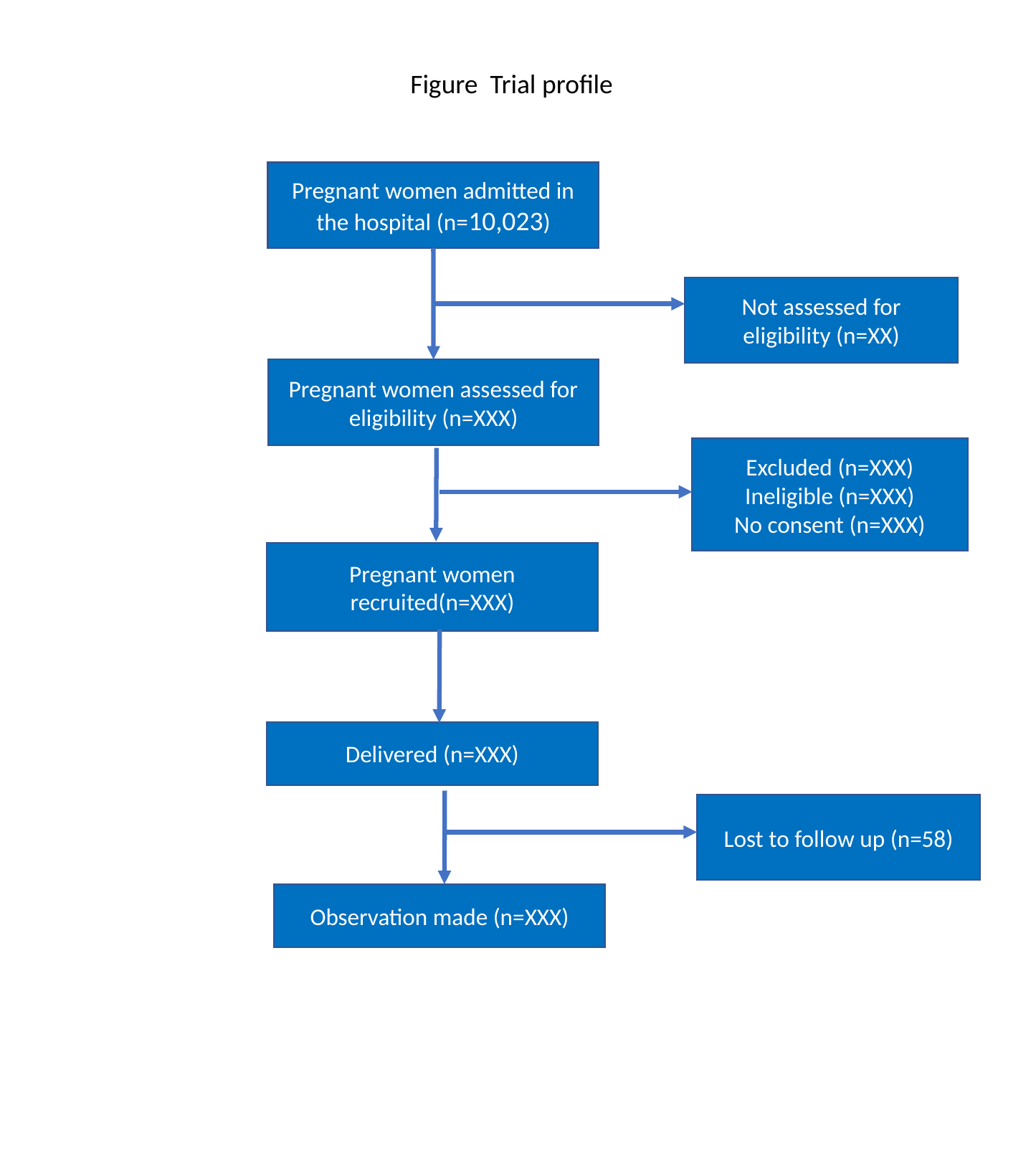

Figure Trial profile
Pregnant women admitted in the hospital (n=10,023)
Not assessed for eligibility (n=XX)
Pregnant women assessed for eligibility (n=XXX)
Excluded (n=XXX)
Ineligible (n=XXX)
No consent (n=XXX)
Pregnant women recruited(n=XXX)
Delivered (n=XXX)
Lost to follow up (n=58)
Observation made (n=XXX)
