# Supplementary material for: REFINE (Rapid Feedback for quality Improvement in Neonatal rEsuscitation): an observational study of neonatal resuscitation training and practice in a tertiary hospital in Nepal
Source: BMC Pregnancy Childbirth. 2020 Dec 3;20:756. doi: 10.1186/s12884-020-03456-z (PMC7712979; doi:10.1186/s12884-020-03456-z)
Supplement: Supplementary file 3 — Additional file 3. Indepth interview questionnaires. [file 12884_2020_3456_MOESM3_ESM.docx]

**Process Evaluation of Quality Improvement Package in a Tertiary Hospital of Nepal: REFINE**

**Semi-Structured Guideline for Health Staffs on NeoBeat Newborn Heart Rate meter**

| **Innovation** |
| --- |
| - What is the evidence around use of heart rate for improving cardiovascular physiology? - What does heart rate indicate you about the condition of the newborn? - What is the current resuscitation protocol? - What is the current understanding of heart rate in relation to resuscitation? - When is it important to measure HR? |
| - Could you explain to me what NeoBeat is? (Want to see if interviewee understands the concept of NeoBeat) - Are you familiar with the NeoBeat used in Hospital? If yes, where is it used and why do you think it is used?   ***Probe:*** (*Is it on placed on all babies or only on not crying babies?)Does it play any role in resuscitation? What about the safety issues? Ergonomics? What is the value of NeoBeat?* |
| - What method did you use before NeoBeat to obtain heart rate information?   ***Probe:*** *Cord palpation, stethoscope, etc. How long did you measure heart rate in one time?*   - Has use of NeoBeat changed your clinical routine?   ***Probe:*** *Has it changed the way you provided resuscitation to non-crying/non-breathing babies? Has it made resuscitation effort easier? Explain How?*   - How has NeoBeat affected the neonatal resuscitation protocol? Before and after implementation of NeoBeat? |
| - How do you compare resuscitation methods before and after NeoBeat?   ***Probe:*** *Current and previous time to stimulation and ventilation? What are the newborn outcomes on birth asphyxia before and after? What do you prefer most? The old methods or the new ones with NeoBeat? Why?* |
| - What do you have to say about the reliability and validity of NeoBeat?   ***Probe:*** *Do you feel that NeoBeat provides reliable heart rate and acts properly when put on the baby? Do you feel that it is giving you continuous information on the heart rate when you are ventilating the baby?*   - What do you feel about the fit-to-size of NeoBeat?   ***Probe:*** *How do you reflect on the use of NeoBeat in babies of different sizes?*  ***Probe:*** *LBW, overweight newborns, Have you seen bruises or scratches on the baby after using NeoBeat? How long does it take to put NeoBeat on the baby? How long does it take to display the heart rate after NeoBeat is placed on the baby?*   - Does the display on NeoBeat light up when you pick it up? Are there any problems activating NeoBeat?   (**Specify** in seconds if possible or ask for perception of how fast it goes – Fast/very fast/it takes too long - etc. *If it takes too long why?*   - Have you experienced anything that becomes difficult when using NeoBeat? (delayed intervention, added confusion, hypothermia) Ergonomics |
| **Recipients** |
| - How has the use of NeoBeat helped you professionally?   ***Probe:*** *Do you want to continue using this? When do you use it most? Has the use of device helped in building confidence? Building team spirit?* |
| - How were you trained/oriented on neonatal resuscitation?   ***Probe****: Was any training provided? Was it practice-based or theory-based? Skill Drills? How long (Duration) was the training? How often do you get training?*  ***Probe:*** *What does NeoBeat training look like? Was it e-learning through Video? Mentorship? Group discussion? Posters? Flipchart?*   - Was the training adequate or enough?   ***Probe:*** *In what sense, was the training adequate? What differences were there compared to previous neonatal resuscitation trainings? Was sufficient time given for practice? How capable were the trainers?*   - How were the trainers?   ***Probe:*** *Did the trainers provide you updated knowledge and techniques with rationale? If yes, what was your new learning?* |
| - Were there any additional QI interventions apart from NeoBeat newborn heart rate meter?   ***Probe:*** *What kind of interventions? Plan, Do, Study, Act (PDSA) meetings? Clinical debriefings? On-site coaching for any inconsistencies or gaps in newborn resuscitation process? Skill drills?* |
| - How do you clean NeoBeat? Is it difficult or hard to clean?   ***Probe:*** *How do you know that NeoBeat is cleaned and ready to use? Did anyone teach you about the procedure?*   - What do you do with NeoBeat after it has been used?   ***Probe:*** *Who takes account of its security? Where is it kept? Is it safe there?*   - Has the product experienced any damages in any way? If yes, elaborate on when and what happened   **Probe:** *Was the damage due to ergonomics or due to mishandling?* |
| - What were the barriers to implementing NeoBeat?   ***Probe:*** *Was it difficult to use? Readings not accurate or fluctuation? Hindering resuscitation process? Cleaning and disinfection process: time taken/is it simple or complicated?* |
| **Context** |
| - How many NeoBeat are available for use at hospital now?   ***Probe****: How many NeoBeat are required in the labour room? Is it adequate? Deliveries in month? What is the peak time in a 24-hour period? Season (which months are busier)?*   - How new products are usually introduced in the facility?   **Probe:** *Who takes the charge of it? How is new products allowed to be used?*   - How often do you charge NeoBeat?   ***Probe:*** *Where have you placed the charging stand? How do you get to know about its charging status? How long does it take to charge and how long does it last? How easy is it to read battery indicator? accuracy*   - Where is the resuscitation related equipment placed during resuscitation? - How do you use the HR feedback when you resuscitate?   ***Probe:*** *Does the HR feedback help you resuscitate better? If yes, how?*  ***Probe****: When you ventilate, what kind of feedback do you find useful?*   - When in Confusion while using the device, who is your ‘Go-To-Ask’ person?   ***Probe:*** *Can you explain your experience? How do you find the teaching learning environment?* |
| - How is the support from the unit in-charge or head of department?   ***Probe:*** *Are they supportive? Do they approve of the benefits or deem it unnecessary or burden?*   - Does the hospital direct or know about it?   ***Probe:*** *If Yes, what does s/he know? What has been his/her perception?* |
| **Facilitation** |
| - How many care providers are you generally when you resuscitate a newborn? Can it be done alone?   ***Probe:*** *How many health staffs have actually been using/ trained and knowhow of the equipment?*   - What are the responsibilities and roles for the (each) care provider during resuscitation? - What are the key things that are being shared between colleagues during resuscitation? |
| - How do you perform your skill drill?   ***Probe:*** *How often? Has it helped in enhancing skill?* |
